# The global impact of COVID-19 on abortion care

**DOI:** 10.1016/j.heliyon.2023.e16094

**Published:** 2023-05-08

**Authors:** Isabella Ong, Aqilah Dariah Mohd Zulkarnain, Kelly Zhi Qi Lim, Daniel Boon Loong Teh, Wilson Tam, Zhongwei Huang

**Affiliations:** a Yong Loo Lin School of Medicine, National University of Singapore, 1E Kent Ridge Road, NUHS Tower Block, Level 11, 119228, Singapore; b Department of Biochemistry, Yong Loo Lin School of Medicine, National University of Singapore, 117456, Singapore; c Alice Lee Centre for Nursing Studies, Yong Loo Lin School of Medicine, National University of Singapore, Level 2, MD 11, 10 Medical Drive, 117597, Singapore; d Department of Obstetrics & Gynaecology, Yong Loo Lin School of Medicine, National University of Singapore, 1E Kent Ridge Road, NUHS Tower Block, Level 12, 119228, Singapore; e NUS Bia-Echo Asia Centre for Reproductive Longevity and Equality, Yong Loo Lin School of Medicine, National University of Singapore, Singapore

**Keywords:** COVID-19, Pandemic, Abortion, SRH, Contraception

## Abstract

**Background:**

The COVID-19 pandemic placed unprecedented strain on healthcare globally, which exacerbated factors leading to unplanned pregnancies.

**Objectives:**

The primary objective was to analyze the effect of COVID-19 on abortion services globally. Secondary objectives were to discuss issues regarding access to safe abortion and provide recommendations on continued access during pandemics.

**Search strategy:**

A search for relevant articles was conducted by utilizing multiple databases (PubMed, Cochrane, etc.).

**Selection criteria:**

Studies on COVID-19 and abortion were included.

**Data collection & analysis:**

The legislation governing abortion services across the globe was examined, inclusive of modifications to service provision during the pandemic. Global data on abortion rates and analyses of selected articles were also included.

**Main results:**

14 countries instituted legislative changes related to the pandemic, 11 relaxed abortion regulations, while three restricted abortion access. An increase in abortion rates was seen particularly where telemedicine was available. Where abortions were postponed, second-trimester abortions increased after services resumed.

**Conclusions:**

Legislation, risk of exposure to infection, and access to telemedicine affect access to abortion. The use of novel technologies, maintaining existing infrastructure and enhancing the roles of trained manpower for safe abortion access is recommended to avoid the marginalization of women's health and reproductive rights.

## Teaser

**Synopsis**: Legislation, maintaining existing infrastructure and enhancing the roles of trained manpower for safe abortion access is recommended to protect access to abortion.

## 1 Introduction

Infectious diseases such as COVID-19 have crippling implications due to their mode of transmission. The world's connectivity and the proximity of large populations act as catalysts for rapid spread via the respiratory route. With a growing global tally standing at 164 million active COVID-19 cases and 3‧41 million deaths from the pandemic as of May 2021 [1], this pandemic has had an unprecedented impact on modern healthcare systems. Combating the pandemic drained manpower and equipment from other clinical needs, such as sexual health and contraception.

From 2015 to 2019, 73 million out of approximately 121 million unintended pregnancies recorded annually resulted in abortions [2]. The main reasons for abortion can be broadly classified into women-centered (timing, mental and physical health, family planning), other-centered (intimate partner relationship issues, existing childcare responsibilities) and material reasons (financial, housing limitations) [3]. However, to contain the spread of COVID-19, countries have enforced lockdowns, which could exacerbate intimate partner and sexual violence that may lead to unwanted pregnancies [4,5]. Many individuals also faced financial difficulties due to poor economic outlook and job losses [6].

In certain areas, contraception and abortion have been classified as non-essential services due to resource shortages, such as lack of personal protective equipment (PPE) and manpower, which were prioritized for COVID-19 facilities. Women then faced barriers in accessing abortions, resulting in many considering unsafe abortions [7].

With a global pandemic that disrupts access to reproductive healthcare, there is an undeniable risk to women's health. The primary objective of this systematic review is to analyze the published literature on the effect COVID-19 had on abortion services globally, and abortion services refer to the provision of information, abortion management which includes a medical procedure that can be effectively managed by health workers using medication or a surgical procedure appropriate for the gestational age, as well as post-abortion care. The secondary objectives are to discuss issues with access to contraception and safe abortion, and to provide recommendations on ensuring safe access to abortion services during a pandemic.

## 2 Materials & methods

Our systematic review search was completed on February 18, 2021. Various databases were searched, namely, PubMed, Embase, Medline (Ovid), Applied Social Sciences Index & Abstracts (ProQuest), Cochrane, Web of Science, and Scopus. We intentionally include the following search terms to encompass all articles which discussed about abortions/unplanned pregnancies in relation to the pandemic and this included: Pandemic AND Abortion, COVID-19 AND Abortion, Pandemic AND Unplanned Pregnancy, COVID-19 AND Unplanned Pregnancy, Unplanned Pregnancy AND H1N1, and lastly Pandemic AND Termination of Pregnancy.

We only included studies on pandemics involving acute respiratory viruses and studies on abortion (termination of pregnancy) but not spontaneous abortion (referring to spontaneous pregnancy failure) as this were unprecedented health events which would needed to be treated and managed, different from abortion services which a woman would search out of her own volition. Studies on HIV/AIDS were excluded as the World Health Organization (WHO) has defined it as an epidemic. Studies solely about either pandemics or abortion were excluded as these studies only discuss on either topic alone. As there were no articles on pandemics and abortion other than COVID-19 during our detailed search, the focus on the manuscript was examining how the pandemic COVID-19 altered policies/healthcare approach resulting in the change on how abortion services were performed and impact on couples with fertility issues. Importantly, we also wanted to examine the existing legislature governing abortion services throughout the world and how these changed during the pandemic to examine on the provision and access to abortion and contraceptive services to women. The results were independently screened for eligibility by three reviewers (IO, KL, AD) based on the titles, abstracts, and keywords. Full texts of the potential eligible articles were retrieved and screened. Key information of each study article was compiled. The data was extracted by three reviewers (IO, KL, AD) after a common consensus was reached. When a consensus could not be reached, a fourth reviewer (HZ) would be consulted. Relevant content from the articles was then analyzed and put together in a narrative manner.

We also performed a search of all available published articles on the legislations governing abortion in countries worldwide as abortion services must be legalized to be carried out safely in countries by well-trained healthcare providers. This was performed by searching within the WHO's Global Abortion Policies Database and the Guttmacher Institute's abortion factsheets [8,9]. These sources were valuable to ensure that all data on legalizing abortion were authentic and corroborated with governmental websites where available.

Data on abortion rates was obtained by searching various online governmental databases.

## 3 Results

**Fig. 1:**
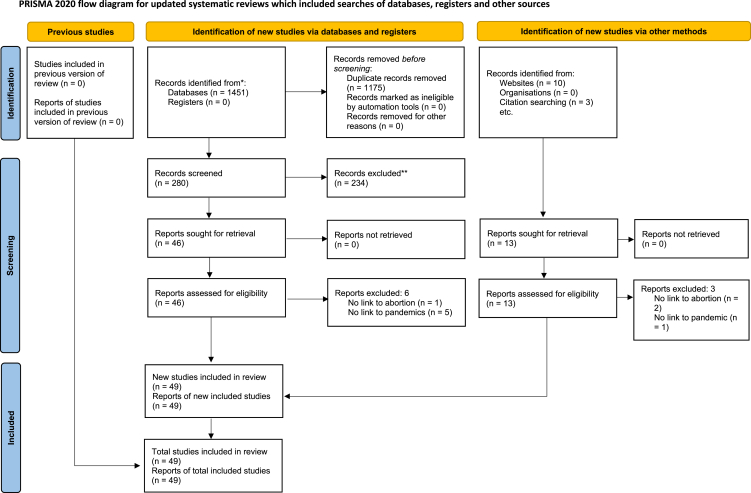
PRISMA Flowchart of systematic review

Our search identified 1451 articles from electronic databases and 13 from other sources, with 1175 articles excluded during the initial title and abstract screening, 234 duplicates were removed, and 9 articles were excluded in full-text screening (see Fig. 1: PRISMA Flowchart). Forty-nine articles were selected – all were in English, except one in Chinese. The articles were sorted according to the country of origin (see Table S1 in supplementary material).

### 3.1 Approaches to abortion

**Fig. 2:**
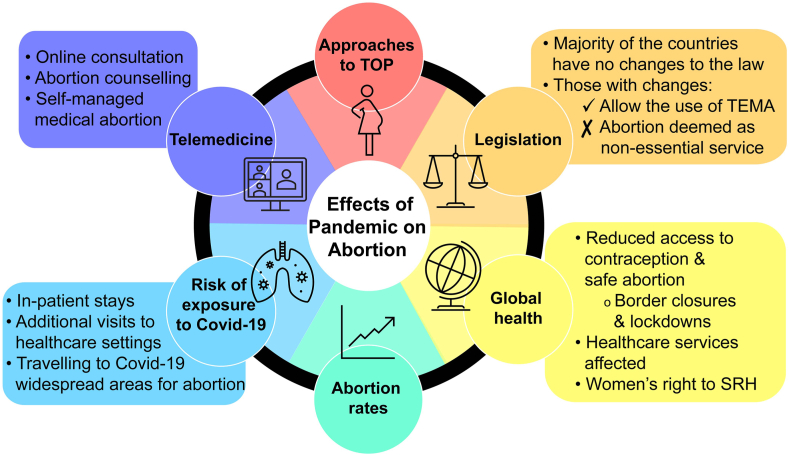
The Effects of Pandemics to Abortion Care

The various approaches to abortion are summarized in Fig. 2.

An important issue addressed by four articles was the implication of COVID-19 in pregnancy. Thus far, COVID-19 infection had not been shown to increase risk of adverse maternal outcomes, [S10] or adverse fetal outcomes. [S6] Abortion was not indicated in COVID-19 patients as infection would not had adverse outcomes on the pregnancy. [S8] As such, the risk of health consequences consequent to abortion should be minimized even in women infected by COVID-19. [S23].

Two articles examined the need for sexual and reproductive health (SRH) services during the pandemic. An Indian cross-sectional study was circulated via Whatsapp to mostly urban-residing women. One-fifth of participants believed that COVID-19 infection in pregnancy would require abortion, [S12] demonstrating the general population's flawed understanding of COVID-19's impact on pregnancies. A Chinese cross-sectional study showed that 8.9% experienced difficulties in accessing contraception, which could lead to increased abortion requests. [S7].

### 3.2 Legislation

**Fig. 3:**
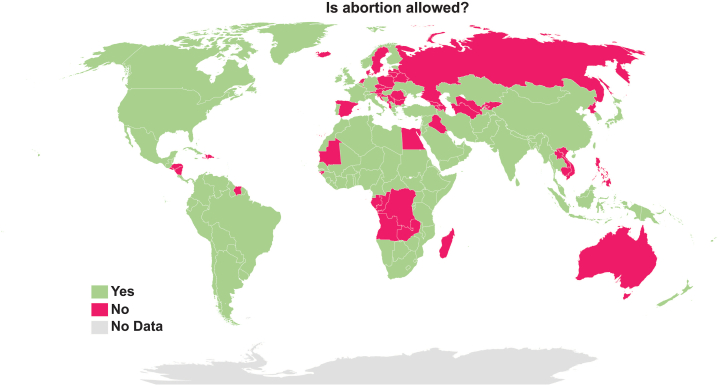
The legality of abortion globally. The detailed description of abortion legislature can be found in Table S1.

**Fig. 4:**
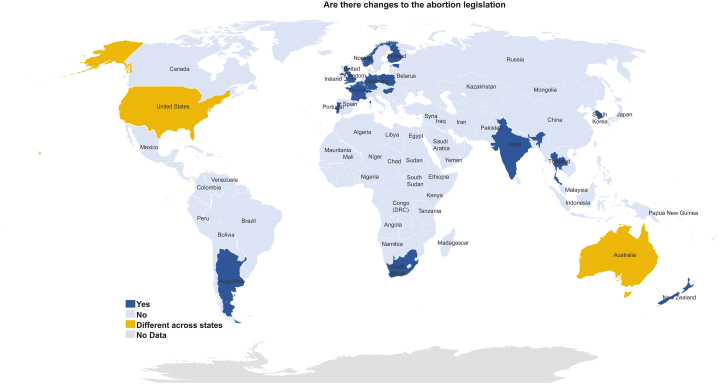
Changes to legislature governing abortion care during the pandemic

**Fig. 5:**
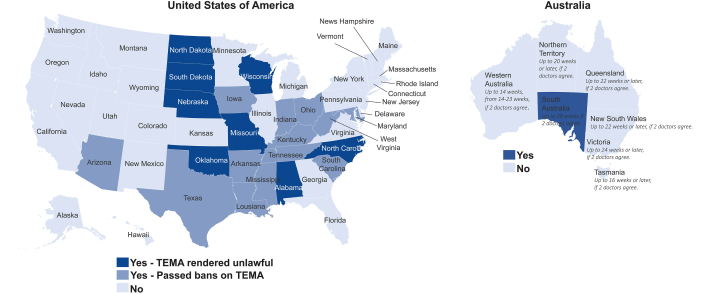
Changes to legislature governing abortion care during the pandemic in the USA and Australia. The states in these countries have different abortion legislations

Access to abortion is mandated under international human rights law. [S4] Twenty-four articles discussed the changes in legislation. Relevant information regarding abortion legislation was put into tables (Tables S2, S3, S4, S5, S6, S7) which can be found in the supplementary material. This is also supplemented by infographics illustrating abortion regulations globally (Fig. 2, Fig. 3, Fig. 4, Fig. 5).

Six articles discussed the legality of telemedicine. Telemedicine Early Medical Abortion (TEMA) was introduced to tackle abortion care which is time-sensitive, as most countries stipulated gestational limits for abortion. This comprised of a tele-consult with a trained healthcare professional before the administration of mifepristone, followed by misoprostol 24–48 h later. [S24] Australia, Canada, China, Nepal, South Africa, Scandinavia, [S2] France, [S17] United Kingdom (UK), [S27] and certain states in the United States of America (USA)[S49] utilized TEMA to reduce disruption to safe abortion services. France, Finland, and Scotland, [S47] had also increased gestational limits for medical abortions.

Twenty-one articles showed the restriction on abortion services by banning telemedicine or postponing abortion. TEMA had been blocked or banned in Austria, Cambodia, Ghana, Iceland, Italy, Spain, Tunisia, [S25] and some states in the USA, [S31, S40–S41] through bans on telemedicine consults for abortion and mail delivery of mifepristone. [S36] Brazil, [S3] Hungary, [S47] Italy, [S18] and certain USA states, [S29, S32] postponed abortion services. These were categorized as “non-essential” or “elective procedures”; [S39, S42, S44] which were halted to divert resources to deal with the pandemic. [S33].

In the USA, 70% of abortion clinics surveyed cancelled or postponed appointments. [S34] Visits to abortion clinics were reduced by 32%, with additional 23% decrease where elective surgical procedures were banned. However, there was no statistically significant decrease where surgical abortions were explicitly banned. [S35] Hence, it was unlikely that demand for abortion decreased even when banned, as abortion rates remained the same. [S9] Since legislation did not reduce abortion rates, policies ensuring access to safe abortion must be implemented to prevent adverse maternal outcomes associated with unsafe abortions or women having to carry unintended pregnancies to term. [S27, S46].

Table 1 summarizes the changes in abortion legislation by 19 countries during the COVID-19 period. A critical challenge faced was ascertaining changes resulting from the pandemic directly and changes that occurred coincidentally during the same period. The changes in legislation in the first five countries were coincidental in terms of timing to the pandemic as changes to the legislation were ongoing prior to the pandemic. The next 11 relaxed abortion legislations, while the last three restricted abortion services due to issues arising from the pandemic.

**Table 1:**
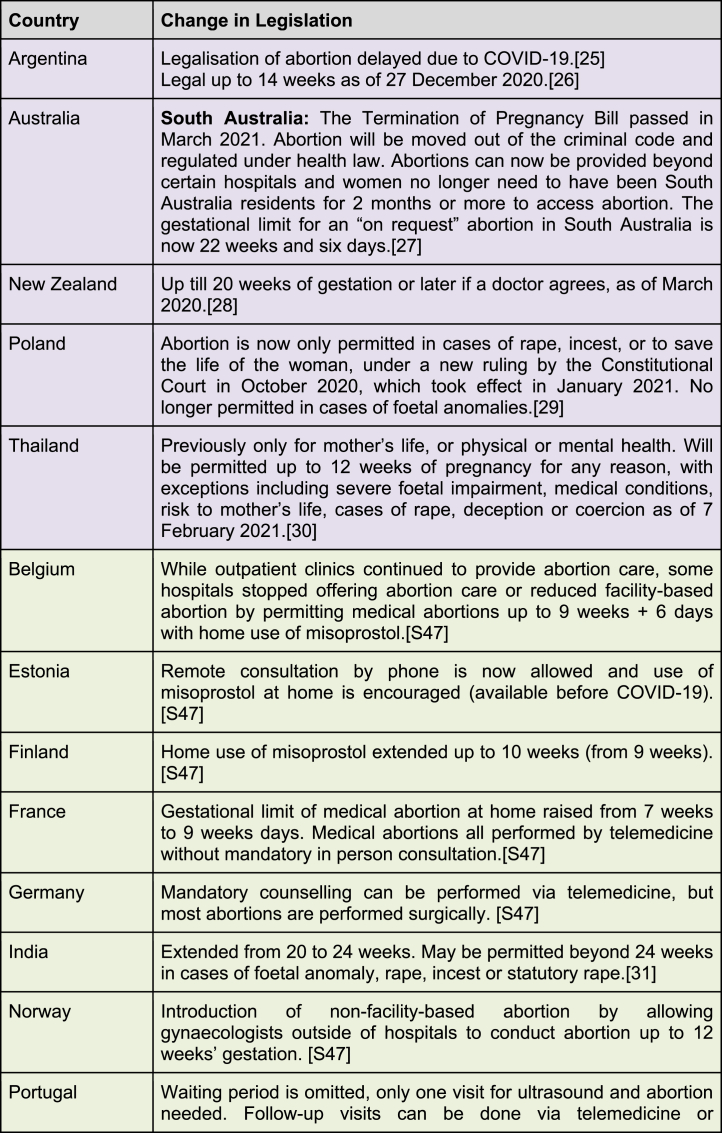

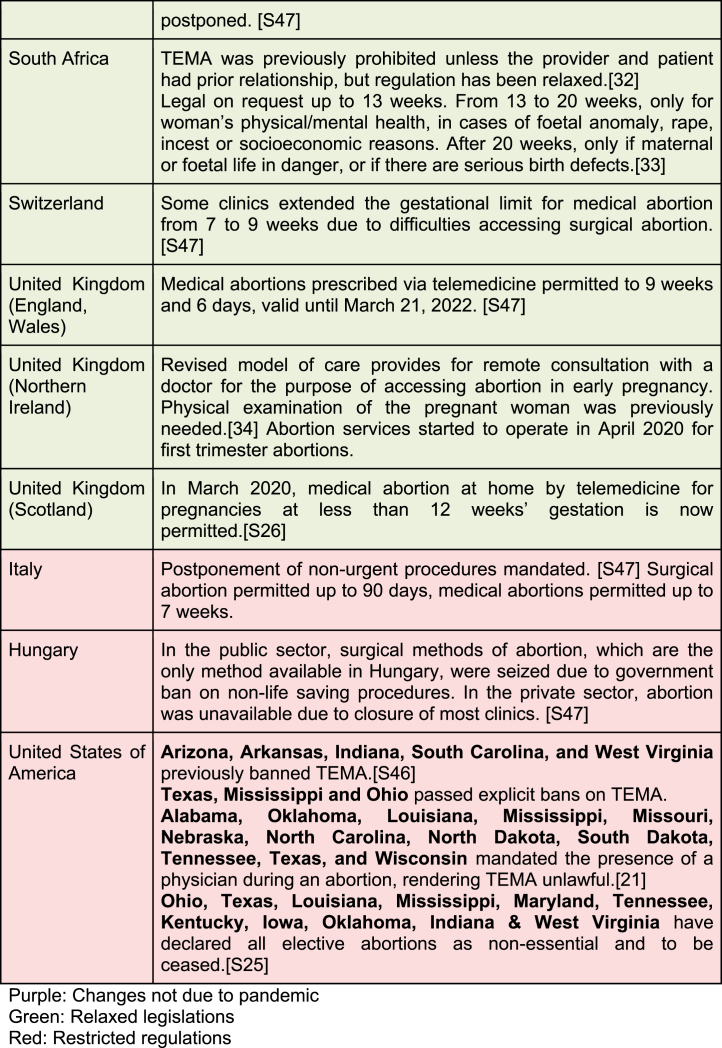
Changes in abortion legislation [[10], [11], [12], [13], [14], [15], [16], [17], [18], [19]].

### 3.3 Risk of exposure to COVID-19

Thirteen articles discussed the risk of exposure to COVID-19 infections when in-person consults for abortion care were required. Four articles raised concerns on potential increased risk of nosocomial infection due to abortion regulations. In Italy, medical abortions comprise only 20% of abortions, and was an outpatient procedure in only five out of 20 regions [S19]. Hence, most abortions are performed in the inpatient setting. In the USA, certain areas mandate two clinic visits for abortion, which increases patients’ exposure to healthcare environments [S41]. A case report on surgical abortion in the USA concluded that prolonged hospital stay causing hospital resource strain could increase the risk of COVID-19 infection [S30]. Therefore, limiting hospitalization and discharging patients early was encouraged [S3].

Eight articles mentioned that there would be more harm when safe access to abortion care was compromised. About one-third of women seeking abortion care in India reported difficulties accessing abortion care due to fear of contracting COVID-19. [S16] One-third of women surveyed in the USA reported having abortion clinic appointments delayed or cancelled. [S35] Women also reported difficulties in visiting clinics because of childcare and transport disruptions. [S37] This resulted in travelling further for abortion services, [S29] keeping an unintended pregnancy, [S28] or resorting to unsafe abortions e.g. herbs or illegal medication. [S40] Women who travelled seeking abortions risked violating stay-at-home orders. [S39] Unsafe abortions or continuing pregnancies would further strain existing healthcare resources if complications arose. [S9] Hence, a balance between provision of abortion services and managing healthcare services during the pandemic must be enforced.

### 3.4 Global health & rights

Seven articles commented that access to SRH and abortion services is a global health and human rights issue. Removing barriers that deny access to abortion would uphold the rights to reproductive health. [S5].

Médecins Sans Frontières called for increased resources for self-managed and community-based SRH services. [S21] Marie Stopes International (MSI) also spotlighted this crisis as an opportunity to expedite change and innovate SRH programs which would mitigate adverse effects secondary to the pandemic. [S22] Universal access to abortion services to prevent unsafe abortions should be guaranteed. [S4].

In places with sub-optimally operating health systems, the pandemic further aggravated limited resources such as trained manpower, logistical and infrastructural support. [S15] COVID-19 restrictions also led to border closures, transport services limitations, [S14] supply chain disruptions and abortion clinic closures. Women in countries with abortion bans like Malta turned to ordering medications online, which could be unsafe. [S20].

Three articles highlighted the redirection of healthcare resources from SRH and abortion care to provide care for COVID-19 patients. [S48] In Italy, even though 70% of doctors were conscientious objectors, many abortion providers were transferred to cover COVID-19 wards. [S18] Across China, 21,569 healthcare professionals were redeployed to Hubei province to support the COVID-19 response, which reduced the maternity services workforce. [S11].

Women were disproportionately affected across socioeconomic strata. Lower-income women and women of color comprised 75% of those seeking abortions in the USA. [S36] With certain states restricting abortion access, women had to choose between violating stay-at-home orders, or continuing with unwanted pregnancies. [S39] Women unable to access abortion services could suffer from long-term physical, mental, financial and social consequences, [S42] like financial stress and domestic violence. [S1, S13] As such, a triage framework for SRH services was proposed to ensure access when circumstances warranted judicious use of limited resources. [S44].

### 3.5 Telemedicine

Twenty-six articles recommended the use of TEMA to protect both physicians and patients from the risk of exposure to COVID-19, [S10] decrease inequalities in access, [S2] and reduce the need to travel for abortion care. [S49] In the USA, independent abortion providers reported cancelling or postponing appointments because 40% of patients had confirmed or suspected COVID-19 infections, and 13% due to COVID-19 related travel restrictions. [S34] Hence, telemedicine would prevent delays in care by forgoing in-person visits. [S35–S36].

Telemedicine supported public health responses to curb COVID-19 spread. [S4] As such, there were calls to allow telemedicine to increase access and decrease COVID-19 exposure in Italy. [S18–S19] TEMA was employed to increase access to medical abortion in Australia, Canada, China, France, Nepal, Scandinavia, South Africa, the UK and some states in the USA, [S25] as well as providing contraception, STI prevention, [S48] and screening for intimate partner violence and depression, which had increased in prevalence. [S45].

Three articles illustrated the receptiveness towards TEMA use. In the USA, there was a 27% increase in requests from 27 March to April 11, 2020. Eleven states showed significant increase in requests ranging from 22% in Ohio to 94% in Texas. [S37] Similarly, nearly half of MSI-UK's abortions were delivered at home between 14 April and May 10, 2020. [S22].

Four articles demonstrated the accessibility and efficacy of TEMA. A systematic review of 19 papers showed no significant difference between the success rates of home-based and clinic-based medical abortions. [S38] It received positive feedback from French women in the South and Corse regions, [S17] and in the UK, where 71.3% of the women preferred telemedicine over in-person consultation. [S26] A study in the UK conducted “no-test” protocols, where appropriate patients accessed TEMA. The success rates were 98.8% in the telemedicine-hybrid cohorts, [S24] demonstrating its high safety profile.

However, in India, telemedicine could only be used for history taking and counselling but not for administration of abortion. [S14] Logistical issues like insufficient resources, unclear TEMA guidelines and lack of transport would lead to 26 million couples facing an unmet need for contraception if the situation worsened. [S15–S16].

Another platform where abortion pills could be obtained would be via online services from organizations such as Women on Web and Women help Women. In Malta, travel restrictions during COVID-19 led to increased purchases of abortion pills via online platforms. Therefore, the true rates of abortion could have been much greater than what was recorded. [S20].

Appropriate deployment of no-contact protocols and TEMA would not only allow for timely access to abortion, [S43] but could reduce the risk of disease transmission, use of PPE, [S41] and other resources. [S40] Policymakers should consider a minimum service package to prevent underground abortions, [S27] which could cause death or lifelong disability. [S26] Beyond the pandemic, it would allow greater access to abortion and SRH services. [S38].

### 3.6 Abortion rates

The following abortion rates (Table 2) were obtained from available online data as of May 2021. In Hong Kong, England and Wales, where there was access to TEMA, the abortion rates for the same period in 2020 increased as compared to 2019. In Oregon, the abortion rate decreased. While access to abortion was protected in Oregon [21], women might have chosen to cross state borders due to the distance from in-state abortion clinics, as 78% of Oregon counties did not provide abortion services [22]. Hence, the actual numbers may differ. In Texas, the abortion rate decreased during the same months in 2020, which demonstrated that abortions declined in Texas during the executive order to postpone elective procedures. Abortions ≥12 weeks gestational age increased after the order expired due to delays in abortion care. [S31].

**Table 2:** Data on Changes in Abortion Rates with respect to alterations in regulation, policy and legislations.

Country/State	2019	2020
England and Wales [23], United Kingdom	105,540 (Jan–Jun)	109,836 (Jan–Jun) ↑4%
Hong Kong [24], China	1851 (Jan–Aug)	2155 (Jan–Aug) ↑16%
Oregon [25], USA	8688	5935 ↓31.7% (could be masked by the fact that >75% of Oregon counties did not provide abortion services)
Texas, [S31] USA	18,268 (Feb–May)	16,349 (Feb–May) ↓10.5% (likely attributable to postponed elective procedures)

## 4 Discussion

### 4.1 Main observations

Through this extensive systemic review, we observed that globally, the legislation governing the provision of abortion services was variable and even divided within the same country. This was likely attributable to vast differences in religious, cultural background and beliefs, which during difficult times like the pandemic, could give rise to the de-prioritization or even closing of sexual and reproductive health (SRH) services such as abortion and contraceptive services. When access to SRH services became limited or banned, women would be highly disadvantaged which might lead to more harm than good as illegal and unsafe abortions could take place and further jeopardized women's health and their future reproductive potential. We therefore proposed the following recommendations to emphasize the pertinence of SRH health services, and the priority needed to allow access to such services.

#### 4.1.1 Abortion as a Women's right

According to international human rights law, the right to make decisions regarding one's physical body, and access to healthcare services were vital aspects of human rights. [S4] Evidently, this right was violated by restrictions on abortion services. Women who had to continue with their pregnancies unwillingly or who turned to unsafe methods of abortion could suffer from the negative sequelae. Allowing continued access to abortion as an essential health service not only saves women and is a human right which should be advocated for and protected at all times.

#### 4.1.2 Overcoming barriers to ensure access to SRH and abortion care in the context of a pandemic

Adaption of abortion practices worldwide permitted access despite limitations posed by the pandemic. With increased reports in sexual violence [26], movement restrictions, clinic closures, reduced staffing, and disrupted access to contraception, protecting access to SRH services would minimize the likelihood of unsafe abortions.

The safety of both women and healthcare professionals should be prioritized, hence, contactless models of patient care should be explored. To facilitate this while recognizing inequities in access to technology, a triage questionnaire for tele-consult eligibility could be used. If eligible, satellite clinics equipped with computers and fast-speed connections for tele-consults could be utilized. The medications prescribed could then be dispensed by community health workers (CHWs). This reduces the need for PPE by allowing minimal patient contact. A similar telemedicine triage model had been used for chronic pediatric conditions and was found to increase treatment uptake [27].

Medical abortions were made more accessible due to TEMA, as opposed to surgical abortions, which required more healthcare resources due to hospital admissions. Novel technologies such as using consumer-grade smart watches for real-time remote vitals monitoring during the peri-procedure phase could be utilized in a pandemic to monitor stable patients for complications, with 5G networks increasing the safety profile of remote patient care [28]. However, the use of such devices still requires more validation as the current most common use is for heart rhythm monitoring [29]. These methods would also only be possible in countries which are amenable to such technologies.

However, although uncommon, there were risks associated with abortion such as heavy bleeding, incomplete abortion, and post-abortion infections [30]. Women should be counselled before treatment to recognize complications. An online self-screening questionnaire for symptoms of complications which provides relevant recommendations, such as indications for acute medical care could be given to patients. A similar self-checking tool was used in Singapore for the general public to assess for possible COVID-19 symptoms with recommended steps to take [31]. However, the ability of countries to implement telemedicine services was constrained by funding, infrastructure, regulations, political will, as well as inequities in access.

Access to contraception was pivotal in preventing unplanned pregnancies. However, contraceptive production slowed down or stopped because of either lockdown or safe distancing measures. This, along with disrupted access to trained medical professionals, would cause an estimated additional 7 million unintended pregnancies if continued for 6 months [32]. To illustrate the urgency of this issue, Asia produced the majority of contraceptives, with India producing large volumes of generic contraceptives. However, their Active Pharmaceutical Ingredient (API) supplies came primarily from China. With limited API sources, India placed restrictions on the export of progesterone-containing products [33], which disrupted stocks of contraceptive pills and intrauterine devices (IUD), hence global supplies dwindled [34], which in turn directly affected SRH services. Creating a sustainable stockpile of contraceptives and medical equipment would be imperative. It would also be prudent to advise women to opt for longer-lasting methods of contraception such as IUDs. Patient education booklets on contraception which include essential points on types of contraception and locations of SRH services should be offered to increase patient knowledge of contraceptive options.

#### 4.1.3 Sexual & reproductive health legislation to facilitate timely SRH services to all women

A number of governments enacted legislative changes to abortion care to adapt to the pandemic. The American College of Obstetricians and Gynaecologists (ACOG) and other associations opposed restrictions on abortion in view of the clear implications for women's health [20].

In rural areas, policymakers could utilize trained CHWs by permitting them to provide counselling and administration of medical abortions to maximize healthcare services. Provision of medical abortion by nurses has been demonstrated to be as safe, acceptable and effective as those by doctors in the same setting [35,36] and this practice has been encouraged by the WHO [37]. Hence, such services could be utilized to ensure the accessibility of abortion during pandemics.

In developed countries, telemedicine and novel technologies such as smart watches for real-time vitals monitoring should be permitted by legislation. After being studied and validated in the local context to ensure that they are safe, acceptable, and efficacious, these novel technologies can then help to increase the safety profile of home recovery.

Reviewing the legislations and approaches of abortion service management was pertinent to eradicate hurdles in the provision of safe abortion care. Policymakers could formulate a minimum service package for times of crisis to protect and ensure access to contraception, safe abortion care, obstetric and neonatal care to protect these essential health rights.

### 4.2 Summary

Legislation, lockdowns, risk of exposure to infection, and access to telemedicine affected access to abortion services. Telemedicine was especially useful in areas where women found it inconvenient or unsafe to travel. Reported numbers might not reflect the true rates of abortion worldwide as women could leave home to another location to access abortion services, sought unsafe abortion methods, or postponed abortion care. The use of novel technologies for remote vitals monitoring where feasible, maintaining existing infrastructure and enhancing the roles of trained manpower have great potential to protect women's health and reproductive rights.

### 4.3 Strengths & limitations

This study examined how abortion practices have changed across the globe due to pandemics, and how it may have affected abortion rates. It brought together global data on abortion legislation, and the impacts of the pandemic in this area. Potential strategies that healthcare professionals and policymakers can implement to support women seeking safe abortions had been discussed prior.

The long-term impact of COVID-19 could only be evaluated retrospectively as the situation continues to evolve. There were limited studies that focused on the impact of the pandemic on abortion, and the majority of the papers were from the USA and Europe. Hence, other perspectives may not have been adequately represented. Limited data on abortion rates were available for 2019 and 2020 (during the COVID-19 pandemic), as most countries have an average lag time of one to two years before releasing abortion rates, hence data would be accurate only up to when the manuscript was prepared for publication.

## 5 Conclusion

Current studies demonstrated a rise in demand for abortion services during the pandemic, particularly in areas where telemedicine was available. As such, legislation should protect and ensure access to abortion care, especially in these extenuating circumstances, in order to prevent the regression of policies governing women's reproductive health. Policymakers are strongly advised to ensure continued safe access to abortion services and contraception through prevailing policies and legislature and offer a minimum service package to prevent maternal morbidity and mortality due to unsafe abortions.

By strengthening regulatory frameworks and adopting new innovations appropriately, healthcare systems will be able to withstand future health crises and/or pandemics while avoiding the marginalization of women's health. The long-lasting impact of the pandemic on abortion rates and abortion care could only be better appreciated in the years to come. Future studies should continue to examine the evolution of legislation and trends in abortion care worldwide to uphold women's reproductive rights globally and to ensure the well-being of future generations.

## Author contributions

**Daniel Boon Loong Teh, Wilson Tam, Zhongwei Huang**: Conceived and designed the study; Performed literature review; Analyzed and interpreted the data critically; Contributed materials, analysis tools or data; Wrote the paper. **Isabella Ong, Aqilah Dariah Mohd Zulkarnain, Kelly Zhi Qi Lim**: Performed literature review; Analyzed and interpreted the data; Contributed tables, analyses, or data; Wrote the paper.

## Data availability statement

We have provided all information accurate on the date of manuscript preparation provided in the supplementary data.

## Declaration of competing interest

The authors declare that they have no known competing financial interests or personal relationships that could have appeared to influence the work reported in this paper
