# Supplementary material for: The global impact of COVID-19 on abortion care
Source: Heliyon. 2023 May 8;9(5):e16094. doi: 10.1016/j.heliyon.2023.e16094 (PMC10165865; doi:10.1016/j.heliyon.2023.e16094)
Supplement: Multimedia component 1 [file mmc1.docx]

**Supplementary Material**

**Table S1: Summary of Articles**

| **Name of Paper / Authors / Year** | **Country / Type of Article** | **Status of Abortion** | **Impact on Women's Health** | **Recommendations** |
| --- | --- | --- | --- | --- |
| **How COVID-19 Highlights an Ongoing Pandemic of Neglect and Oppression When It Comes to Women's Reproductive Rights** / Dahlen, H. G., Kumar-Hazard, B., Chiarella, M. / 2020[1] | Australia / Commentary | Increased demand for privately practicing midwives (PPM), which is not matched by systemic support for or recognition of home birth. | Increase in number of unwanted pregnancies, which worsens the risk of domestic violence incidents and financial stress, especially among migrant women. | Universities should offer direct entry midwifery programs to help to increase the retention of midwives, where a nursing qualification is not required first. |
| **The impact of COVID-19 on contraception and abortion care policy and practice: experiences from selected countries** / Bateson, D. J., Lohr, P. A., Norman, W. V., et al / 2020[2] | Australia, Canada, China, France, Sweden, UK, USA / Editorial | Increased access via telemedicine in the United States, Canada, United Kingdom, France, Australia, Scandinavia, China, South Africa and Nepal. Regulatory changes have extended the upper gestational limit from 9 to 10 weeks in Finland, to 12 weeks in Scotland and from 7 to 9 weeks in France. | Reduction in legal barriers to sexual and reproductive healthcare have helped to reduce inequality of access to such services. | Continue to advance sustainable contraception and abortion care policies. |
| **Childbirth, Puerperium and Abortion Care Protocol during the COVID-19 Pandemic** / Trapani Júnior, A., Vanhoni, L. R., Silveira, S. K., Marcolin, A. C. / 2020[3] | Brazil / Practice Guideline | Abortions have been postponed in Brazil. | Patients can only get abortions at a later date, which may affect the method used for abortion. | Limit time spent by patient in hospital by discharging the patient as early as possible, and reduce the postponement as much as possible. |
| **Abortion in the context of COVID-19: a human rights imperative** / Todd-Gher, J., Shah, P. K / 2020[4] | Canada / Commentary | Under the international human rights law, it is the state's obligation to ensure abortion access during COVID-19, such as by enabling self-managed abortion via telemedicine counselling and access to medications. | Providing access to safe abortion would allow women to be able to prevent and/or manage unwanted pregnancies, not only for their own health and well-being, but also to support effective public health responses to prevent and treat COVID-19. | Permitting women to undertake safe self-managed abortion with telemedicine counselling, is not simply about harm reduction; it is a human rights imperative. |
| **Why self-managed abortion is so much more than a provisional solution for times of pandemic** / Assis, M. P., Larrea, S. / 2020[5] | Canada / Commentary | Feminist activists provide access to abortion through different means such as safe abortion hotline and telehealth services that delivers pills. | Women should be able to decide how they want their abortion without the fear of judgement. | People should embrace the power of self-management beyond the pandemic. |
| **[Analysis of the pregnancy outcomes in pregnant women with COVID-19 in Hubei Province]** / Zhang, L., Jiang, Y., Wei, M., et al / 2020[6] | China / Comparative Study | Abortion for pregnant women with COVID-19 has no significant impact on the baby, but may improve maternal treatment outcomes. | Determining whether to continue pregnancy when the mother contracts COVID-19, as it has an impact on the treatment outcomes for the mother. | Timely termination of pregnancies may be required to improve maternal COVID-19 treatment outcomes. |
| **Impact of the COVID-19 Pandemic on Partner Relationships and Sexual and Reproductive Health: Cross-Sectional, Online Survey Study** / Li, G., Tang, D., Song, B., et al / 2020[7] | China / Cross-sectional Study | Difficulty in accessing abortions, related to making appointments. | Shortage of contraception and difficulties in access to abortion reduce women's sexual autonomy. | Protect basic sexual and reproductive healthcare services and supplies from disruption during a pandemic. |
| **Is termination of early pregnancy indicated in women with COVID-19?** / Wu, Y. T., Li, C., Zhang, C. J., Huang, H. F. / 2020[8] | China / Letter to editor | Abortion is not indicated in mothers who have contracted COVID-19 except when maternal health is at risk. | Pregnant women who have contracted COVID-19 can continue their pregnancies with close monitoring for hypoxaemia to protect maternal safety. | Monitor pregnant women with COVID-19 closely to determine if there are risk factors that may indicate abortion. |
| **Post Abortion Care and Management After Induced Abortion During the COVID-19 Pandemic: A Chinese Expert Consensus** / Wang, Y., Yang, Q., / 2021[9] | China / Review | Abortion rates are similar whether access to abortion is freely available or restricted. In cases of restricted access, women tend to resort to unsafe abortion outside medical regulation, which is likely to be detrimental to the health of the woman and require more specialist care from the healthcare system. | The post-abortion care (PAC) work due to the COVID-19 pandemic should not be delayed to prevent the repeated threat of abortion in women of childbearing age. The PAC education should emphasize the importance of long-term contraception and its effectiveness to avoid unintended pregnancies. | Healthcare workers and medical staff should be trained to properly protect themselves and others from nosocomial infection, and to prevent or limit the spread of infections in healthcare settings. Awareness regarding COVID-19 virus as well as public education should be implemented. Maximize the use of remote consultations (e.g., via video or telephone) to provide follow-up instructions related to post- abortion care and assessment. |
| **Pregnancy and Perinatal Outcomes of Women with Coronavirus Disease (COVID-19) Pneumonia: A Preliminary Analysis** / Liu, D., Li, L., Wu, X., Zheng, D., Wang, J., Yang, L., Zheng, C. / 2020[10] | China / Preliminary Analysis | Pregnancy does not aggravate mild cases of COVID-19; hence abortion is not indicated. | Pregnant women with mild cases of COVID-19 may continue their pregnancy without increased risk of adverse maternal outcomes. | Evaluate for necessity of anti-viral drug usage in pregnant women with COVID-19 to reduce risks to the foetus. |
| **Sexual and reproductive health (SRH): a key issue in the emergency response to the coronavirus disease (COVID- 19) outbreak** / Tang, K., Gaoshan, J., Ahonsi, B., et al / 2020[11] | China / Commentary | The absence of health care workers from their original duty may still have caused disruptions in regular provision of services, including safe abortions. | Demands of safe abortion services, including information provision, has increased in the hospitals in nearby Hunan Province of China. | Timely planning and actions for epidemiological research and surveillance of the key vulnerable groups of women and adolescents is needed. |
| **Beliefs related to sexual intimacy, pregnancy and breastfeeding in the public during COVID-19 era: a web-based survey from India** / Sahoo, S., Pattnaik, J. I., Mehra, A., Nehra, R., Padhy, S. K., Grover, S. / 2020[12] | India / Cross-sectional | One-fifth of the participants involved in this study agreed with the belief for high risk of miscarriage / abortion and birth defects with COVID-19 infection. | Most pregnant women are struggling with fear and uncertainty with the risk of transmission of infection to their unborn foetus, which needs to be clarified by their obstetricians. | There is a need to correct the misinformation about pregnancy, sexual intimacy and breastfeeding in the ongoing pandemic. Knowledge should be disseminated in simple understandable knowledge. |
| **COVID 19 era: a beginning of upsurge in unwanted pregnancies, unmet need for contraception and other women related issues** / Kumar, N. / 2020[13] | India / Short Review | Reduced access to abortion due to shortage of essential items and healthcare resources. | Reduced access to abortion and contraception, coupled with increased intimate partner or domestic violence, and travel restrictions or risk of contracting the virus, all have led to an increase in unmet needs for family planning as well as ability to care for own health. | Early recognition and better implementation of sexual and reproductive health services and facilities like family planning and contraceptives, abortion care, maternal and neonatal care should be considered essential even during pandemics. |
| **Family planning and abortion services in COVID 19 pandemic** / Sharma, K. A., Zangmo, R., Kumari, A., Roy, K. K., Bharti, J. / 2020[14] | India / Review | Abortion services continue to be provided by both public and private providers. Teleconsultation should be used to take history and for counselling of patients but prescription of medical abortion pills needs direct non-virtual consultation. | Access to abortion may be affected by delayed presentations by the woman due to lack of transport services. | There is a need to change from direct consultation to remote consultation by telemedicine when providing routine family planning services. |
| **Impact of COVID-19 on family planning services in India** / Vora, K. S., Saiyed, S., Natesan, S. / 2020[15] | India / Commentary | Guidelines released by the Ministry of Health recommend continuation of routine reproductive health services. However, the lack of transportation is a barrier to access of such services. | The reduction in abortions performed is about 28%. The numbers of injectable contraception first doses given have decreased by 36%. IUD insertion has shown a 21% decrease. This shows that there is limited family planning service provision and increase in unmet need for family planning. | Health system responses should include delivery of essential services while maintaining physical distancing, use of technology such as telemedicine, virtual appointments, and involvement of the private sector, including for-profit and not-for-profit entities for service provision. |
| **Preparing for an Increased Need for Abortion Access in India during and after COVID‐19: Challenges and Strategies** / Chandrasekaran, S., Diamond-Smith, N., Srinivasan, K.,  Dalvie, S. / 2020[16] | India / Commentary | Telemedicine Practice Guidelines were introduced, but safe abortion services were not specifically identified as approved services. | Reduced mobility, lack of clarity about abortion as an essential service and as a service permitted by telemedicine, shortages in raw material, limited inter‐ and intra‐ state transport of drugs, and additional delays to care are factors that may lead to reduced access to abortion services and drugs. Increased demand for abortion could be a result of increased unintended pregnancies due to the lack of availability of long-acting and permanent methods of contraception, increased intimate partner violence and lack of mobility due to the lockdown. | In addition to strengthening second‐trimester provision of abortion care, efforts should be made to ensure that access to evidence‐based information and care are within reach for anyone who might seek an abortion, irrespective of their gestational age. Safe abortion provision can be expanded to include the cadre of health workers to increase access to safe abortion, especially in rural areas. |
| **COVID-19 impact in abortions’ practice, a regional French evaluation** / Gibelin, K., Agostini, A., Marcot, M., Piclet, H., Bretelle, F., Miquel, L. / 2021[17] | France / Original Research | The French National Health Agency (Haute Autorité de Santé) has urgently recommended the use of medical abortion at home between 7 and 9 weeks of gestation and telemedicine for medical abortion consultations. | The aim of this action was to not exceed the legal limit time for abortion for women, while limiting the exposure of women and healthcare professionals to COVID-19 and preserving medical capacity of health establishments. | The emergency measures implemented by the French National Health Agency (Haute Autorité de Santé) for medical abortion are approved and followed by the majority of health workers performing abortions in the South and Corse regions. This measure may be extended out of the COVID-19 epidemic. During the COVID-19 epidemic, England, Wales and Scotland promoted the avoidance of the use of ultrasound to determine gestational age. |
| **COVID-19 and abortion: The importance of guaranteeing a fundamental right** / Cioffi, A., Cioffi, F., Rinaldi, R. / 2020[18] | Italy / Commentary | Suspended by hospitals due to measure mandating postponement of services that are non-urgent / can be carried out more than 10 days later. | Decreased access to abortion as pre-COVID, 70% of doctors in Italy were already conscientious objectors. With COVID-19, doctors that perform abortions have been transferred away or are in different regions. | Allow abortion to be performed as an outpatient procedure & utilise telehealth. |
| **Safe abortion amid the COVID-19 pandemic: The case of Italy** / Bellizzi, S., Ronzoni, A. R., Pichierri, G., Cegolon, L., Salaris, P., Panu Napodano, C. M., Fiamma, M./ 2020[19] | Italy / Brief Communication | Medical abortions comprise only 20% of TOP methods, and is outpatient procedure in 5 out of 20 regions. | Patients and healthcare staff have increased exposure to COVID-19 due to requirements of inpatient stays. | Allow self-managed medical abortions. |
| **Abortion in the time of COVID-19: perspectives from Malta** / Caruana-Finkel L. / 2020[20] | Malta / Scholarly Journal | Complete ban on abortion (pre-COVID-19). COVID-19’s travel restrictions have led to more women calling for help from abortion support groups and many ordering medical abortion pills online. | Increased anxiety, may cause women to resort to unsafe abortions. | Reform of abortion laws, facilitation of access to abortion. |
| **Now is the time: a call for increased access to contraception and safe abortion care during the COVID-19 pandemic** / Kumar, M., Daly, M., De Plecker, E., Jamet, C., McRae, M., Markham, A., Batista, C. / 2020[21] | Médecins Sans Frontières / Commentary | Access to SRH services have been limited, with shutdowns and delays of such services. | Women may resort to unsafe abortions, leading to preventable deaths or lifelong disability. | Strengthen access to safe abortion and contraception everywhere by engaging with women and their communities to come up with the self-managed models of care. |
| **Reproductive health under COVID-19 - challenges of responding in a global crisis** / Church, K., Gassner, J., Elliott, M. / 2020[22] | Marie Stopes International / Scholarly Journal | Increased access via telemedicine. The implementation of telemedicine was successful, and nearly half of MSI-UK’s abortions were delivered “at home” between 14th April and 10th May 2020. | The impact of service reductions between March and August 2020 suggest there’s an estimated 1.3 million unintended pregnancies in the 37 countries where MSI works this year, which could mean an additional 1.2 million unsafe abortions and 5000 pregnancy-related deaths. | Recommend move for telemedicine globally, but the move towards self-care and telemedicine for abortion as seen in the UK may be restricted by national policy and lack of regulatory approvals in many other countries. |
| **Current State of Knowledge About SARS-CoV-2 and COVID-19 Disease in Pregnant Women** / Gujski, M., Humeniuk, E., Bojar, I. / 2020[23] | Poland / Review Article | Special care should be taken when deciding on abortion. | Minimise the risk of subsequent health consequences. | Further analysis is needed of the incidence of COVID-19 among pregnant women and its consequences. |
| **Effectiveness, safety and acceptability of no‐test medical abortion (termination of pregnancy) provided via telemedicine: a national cohort study** / Aiken, A., Lohr, P. A., Lord, J., Ghosh, N., Starling, J./ 2021[24] | UK / Cohort Study | By 30 March 2020, all the governments in Great Britain had issued emergency legal orders to allow mifepristone to be used at home along with misoprostol up to 10 weeks’ gestation. These approvals permitted abortion providers to implement a fully telemedical service delivery model, including ‘no test medical abortion’ and direct‐to‐patient delivery of abortifacient medications. | Compelling evidence from 52 142 women shows no‐test telemedicine abortion is safe, effective and improves care. | Incorporating no‐test telemedicine into the care pathway is not inferior to the traditional pathway where all patients are seen in person and have an ultrasound scan. Given the advantages of improving access to care, especially in vulnerable groups and in resource‐poor healthcare systems or where patients have to fund their own care, the evidence is compelling that no‐test telemedicine should become routine in the provision of abortion care. |
| **Legal and policy responses to the delivery of abortion care during COVID-19** / Romanis, E. C., Parsons, J. A. / 2020[25] | UK / Letter to editor | Telemedicine-facilitated abortions have been indirectly blocked or outright banned in Austria, Cambodia, Ghana, Iceland, Italy, Spain, Tunisia, and some states in the USA (including Arizona, Arkansas, Alabama, Alabama, Indiana, Louisiana, Mississippi, Missouri, Nebraska, Oklahoma, South Carolina, South Dakota, Texas and West Virginia). | Perpetuation of abortion stigma, risks of unsafe abortions. | Protect access to abortion, especially in places deeming the service as non-essential. |
| **Telemedicine medical abortion at home under 12 weeks’ gestation: a prospective observational cohort study during the COVID-19 pandemic** / Reynolds-Wright, J. J., Johnstone, A., McCabe, K., Evans, E., Cameron, S. / 2020[26] | UK / Original Research | Medical abortion at home by telemedicine for pregnancies at less than 12 weeks’ gestation. | Most women (71.3%) would choose a telemedicine consultation again rather than an in-person visit. | Telemedicine medical abortion at home in the first trimester without routine ultrasound is effective, with low complication rates and high acceptability. Telemedicine service models need flexibility and resources to accommodate those women requiring clinical review post-abortion including ultrasound assessment. |
| **Women are most affected by pandemics - lessons from past outbreaks** / Wenham, C., Smith, J., Davies, S. E., et al / 2020[27] | UK / Commentary | In England, legislation was altered to allow medical abortion at home by using pills after online consultation.  However, in Texas, Ohio, Iowa, Oklahoma and Alabama, access to abortion has been restricted and deemed non-essential. | COVID-19 has caused restrictions on abortions, but this does not limit the demand for its services. More women are seeking underground abortion services which become unsafe. | Medical abortions should be permitted at home, along with online consultation with a physician.  Policymakers should develop a service initial package to be implemented at the start of every pandemic, which ensures access to safe abortion care. |
| **Abortion during the Covid-19 Pandemic - Ensuring Access to an Essential Health Service** / Bayefsky, M. J., Bartz, D., Watson, K. L. / 2020[28] | USA / Perspective | Governors in Texas, Louisiana, Mississippi, Alabama, and Oklahoma have ordered or supported the cessation of both medication and surgical abortion.  Temporary restraining orders (TROs) have blocked state bans while litigation proceeds in Ohio, Alabama, and Oklahoma. | Thousands of women have been refused access to abortion while the state bans were in effect. Without access to abortion, either unsafe abortion or remain pregnant, both of which may require further healthcare services & PPE use. | For the medical profession to speak with a unified voice on several topics, including access to abortion care, as its status is vulnerable despite it being an essential and time-sensitive procedure. |
| **Access to later abortion in the United States during COVID-19 - challenges and recommendations** / Ruggiero, S., Brandi, K., Mark, A., et al / 2020[29] | USA / Commentary | 13 states have attempted to halt abortion services by deeming abortions “non-essential” or “elective” procedures. | Women have to travel long distances to receive later abortion care. | Abortion providers can increase the gestational age up to which they provide services by consulting with other providers experienced in providing later care. |
| **A hospital-based COVID-19 abortion case in the early phase of the pandemic** / Fang, N. Z., Castaño, P. M., Davis, A. / 2020[30] | USA / Case Report | Continued providing abortion care, and advised postponing non-urgent surgery beginning March 16. | To decrease exposure to COVID-19 when seeking surgical abortions. | For safe abortion practices to be mandated whether for patients or clinicians. |
| **Changes in Abortion in Texas Following an Executive Order Ban During the Coronavirus Pandemic** / White, K., Kumar, B., Goyal, V., Wallace, R., Roberts, S. C. M., Grossman, D. / 2021[31] | USA / Research Letter | Abortions declined in Texas during the executive order. Texas facilities provided 18 268 abortions from February through May 2019 and 16 349 abortions during these months in 2020. | Abortions at 12 weeks’ GA or more increased after the order expired, which likely reflects delays in care among those who waited for an appointment and facilities’ limited capacity to meet backlogged patient need. | Although abortions later in pregnancy are very safe, they are associated with a higher risk of complications and may require additional visits com- pared with those provided earlier in pregnancy, which suggest that early pregnancy care is vital. |
| **Coronavirus pandemic stirs fight over abortion rights in US** / Tanne, J. H. / 2020[32] | USA / Journal Article | On 22 March, the governor of Texas, Greg Abbott, signed an executive order banning non-essential medical procedures, of which abortion was deemed non-essential. The ban expired on 22 April the governor issued a new order which allowed abortion care to be fully restored in Texas. The states of Alabama, Arkansas, Iowa, Louisiana, Ohio, Oklahoma, Tennessee also issued bans. (In the US, states can regulate abortions.) Except for Arkansas, where a ban is still in effect, these other bans have been partly or completely suspended by the courts, but the situation remains in flux. | When the ban was in action, women are forced to either delay or travel out of state to access abortion care. Some women even turned to herbs or illegal abortion methods. | To allow abortion rights even during the pandemic. |
| **COVID-19 Abortion Bans and Their Implications for Public Health** / Jones, R. K., Lindberg, L., Witwer, E. / 2020[33] | USA / Commentary | As of May 12, 2020, at least 11 states have attempted to restrict access to abortion by deeming it to be nonessential. These include Alabama, Alaska, Arkansas, Iowa, Kentucky, Louisiana, Ohio, Oklahoma, Tennessee, Texas and West Virginia. | In states where there are abortion bans, women have to travel out of state for the procedure, or continue their pregnancy and hope that they can access abortion care when the ban is no longer in place or attempt to self-manage their abortion (buying abortion drugs on internet). | Governors, public health departments, and the Food and Drug Administration need to have the political will to ensure that abortion care remains safe and accessible for everyone. |
| **COVID-19 and Independent Abortion Providers: Findings from a Rapid-Response Survey** / Roberts, S. C. M., Schroeder, R., Joffe, C. / 2020[34] | USA / Original Research | Cancelation or postponement of abortion services and temporary closure of clinics varied according to whether states in the US considered abortion an essential service. More than 70% of clinics in states that had explicitly declared abortion to be a nonessential service had cancelled or postponed appointments for one or more types, compared with 10–20% of clinics in states that classified abortion as an essential service. | This study found that abortion clinics and abortion patients—in the South (and in some cases the Midwest) not only did not receive the government support that other health care facilities received during the pandemic but were impacted by government responses that explicitly targeted abortion care. | Independent abortion clinics have been deeply affected by the COVID-19 pandemic and have experienced significant disruptions to their ability to provide care. Additional support may be needed to build on this short-term resilience to ensure the sustainability of independent abortion clinics and the well-being of their workforce. |
| **COVID-19 Surgical Abortion Restrictions Did Not Reduce Visits to Abortion Clinics** / Andersen, M., Bryan, S., Slusky, D. / 2020[35] | USA / Original Research | There was a 32% decrease in visits to abortion clinics in 2020 compared to 2019, and states that banned elective medical procedures saw an additional 23% decrease. However, explicitly targeting surgical abortion as part of these restrictions did not have a statistically significant effect. | Around one third of women reported that they had reproductive health appointments delayed or cancelled during the pandemic. | One proposal to maintain abortion access while minimising interpersonal contact is increased application of at-home medication abortion. |
| **Covid-19: Women's health campaigners sue FDA over access to medical abortion pills** / Tanne, J. H. / 2020[36] | USA / Commentary | Prohibition on telemedicine prescribing and mail delivery of mifepristone. | Women on low incomes and women of colour, who make up 75% of those seeking abortion, will bear the brunt of FDA's restrictions. Women face risk of COVID-19 infection if they have to travel to pick up their pills. | Telemedicine use is encouraged and clinicians should be given the flexibility to forgo unnecessary in person encounters. |
| **Demand for Self-Managed Online Telemedicine Abortion in the United States During the Coronavirus Disease 2019 (COVID-19) Pandemic** / Aiken, A. R. A., Starling, J. E., Gomperts, R., Tec, M., Scott, J. G., Aiken, C. E./ 2020[37] | USA / Data Analysis | Increased demand via telemedicine. From 20 March 2020 to 11 April 2020, there was a 27% increase in the rate of requests for self-managed medication abortion across the US. | There is a shift in demand from in-clinic to self-managed abortion likely due to fear of COVID-19 infection or difficulties in visiting clinics because of childcare and transport disruptions | Telemedicine models to be made a policy priority. |
| **Effectiveness, Safety and Acceptability of Medical Abortion at Home versus in the Clinic: A Systematic Review and Meta-analysis in Response to COVID-19** / Gambir, K., Garnsey, C., Necastro, K.A., Ngo, T.D. / 2020[38] | USA / Systematic Review & Meta-Analysis | Home based medical abortion is effective, safe and acceptable to women. There was no difference found between home-based and clinic-based administration of medical abortion in having a successful abortion. | Evidence that home-based medical abortion is effective, safe and acceptable to women is a key strategy to protect access to abortion. | Evidence of safe use of home-based medical abortion should be used to expand women's abortion options and ensure access to abortion for women during COVID-19 and beyond. |
| **How the COVID-19 response is altering the legal and regulatory landscape on abortion** / Ahmed, A. / 2020[39] | USA / Journal Article | Several state governors including Texas, Ohio, Iowa, Mississippi, and Alabama chose to classify abortion as a non-essential or non-emergency service. | Travelling for an abortion due to bans may violate stay-at-home orders and expose women and physicians to illness. | Accessing medical abortion is  necessary in an environment where clinical services may not be available  due to abortion’s status as a non-essential medical service. |
| **Medical Abortion Care During a Pandemic** / Baill, B. C. / 2020[40] | USA / Journal Article | States’ laws do not permit medical abortion counselling and prescriptions via telemedicine. | Endangers the lives of young patients, clinical staff, nurses, and doctors. It may also drive some patients into desperate acts. | Making medical abortion part of telemedicine during this crisis may save the lives of women, nurses, staff, and doctors. |
| **Preserving and advocating for essential care for women during the coronavirus disease 2019 pandemic** / Robinson, E. F., Moulder, J. K., Zerden, M. L., Miller, A. M., Zite, N. B. / 2020[41] | USA / Call to Action | Abortions, whether medical or surgical, have been banned or deemed as non-essential in certain states in the United States. | Increased risk of contracting COVID-19 with current requirements for abortion (mandatory two visits for medical abortions), reduced bodily autonomy if abortions are banned or postponed. | No-test abortions for pregnancies below 77 days should be permitted to reduce risk of contracting COVID-19 and reduce PPE use. |
| **Protecting Access to Abortion During The COVID-19 Pandemic** / Sackeim, M. G. / 2020[42] | USA / Commentary | Surgical and medical abortion services were terminated in some places that deemed abortion as a ‘non-essential’ service.  Texas was the first state to deem abortion as non-essential during the pandemic and ceased abortion services | Women who are unable to access any abortion service due to the termination of services will suffer from long-term physical, mental, financial and social consequences. | Abortion laws during a pandemic should demonstrate more understanding and to protect women's reproductive autonomy. |
| **Reproductive Care During COVID-19 /** McSpedon, C. / 2020[43] | USA / Report | Researchers have developed a no-contact protocol that utilises telehealth for evaluation and follow-up. Abortion medications can also be delivered to patients. | Continue to provide timely access to abortion care. | Essential to consider women and their reproductive health needs in the design process. |
| **Rethinking "Elective" Procedures for Women's Reproduction during Covid-19** / Gross, M. S., Harrington, B. J., Sufrin, C. B., Faden, R. R. / 2020[44] | USA / Essay | Procedures that are non-essential or elective have been postponed or curtailed, and some places have classified abortion as an elective procedure. | Abortions are essential for a variety of reasons, including the physical and mental well-being of the woman. The fight for access to abortion has political roots and goes beyond the restrictions posed by the pandemic. | A triage framework that features multiple dimensions of well-being, nonphysical as well as physical will allow for better prioritisation of services when healthcare resources are in short supply. |
| **Special ambulatory gynecologic considerations in the era of coronavirus disease 2019 (COVID-19) and implications for future practice** / Cohen, M. A., Powell, A. M., Coleman, J. S., Keller, J. M., Livingston, A., Anderson, J. R./ 2020[45] | USA / Clinical Opinion | ACOG and other societies have called for continued access to abortion as essential reproductive healthcare services. | Abortion remains a time-sensitive procedure with substantial risk of harm to a patient if delayed. | Increased use of telemedicine services with increased screening for intimate partner violence and depression using validated questionnaires. |
| **The legal and medical necessity of abortion care amid the COVID-19 pandemic** / Donley, G., Chen, B. A., Borrero, S. / 2020[46] | USA / Original Article | Abortions have been deemed as non-essential in certain states in the United States. | Threats to women's autonomy seem to be politically motivated, as states which are moving to ban or restrict abortion during the pandemic have previously tried to do so, pre-COVID-19, and their motives may not just be related to the pandemic. | Abortion should not be deemed non-essential and should be allowed to continue as it is a time-sensitive procedure. |
| **Abortion regulation in Europe in the era of COVID-19: a spectrum of policy responses** / Moreau, C., Shankar, M., Glasier, A., Cameron, S., & Gemzell-Danielsson, K. / 2020[47] | USA, UK, France, Sweden / Original Research | Refer to Supplementary Table 6: Abortion in Europe. | The lack of a unified policy response to COVID-19 restrictions in Europe has widened inequities in abortion access. | Standardised/Unified policy responses across the European Union to reduce inequities in abortion access. |
| **Centring sexual and reproductive health and justice in the global COVID-19 response** / Hall, K. S., Samari, G., Garbers, S., et al / 2020[48] | USA, UK / Commentary | Sexual and reproductive health providers and clinics may be deemed non-essential and diverted to respond to COVID-19. | Reduced access to family planning and abortion result in increased rates and sequelae from unintended pregnancies, unsafe abortions, complications from pregnancy, miscarriage, sexually transmitted infections, depression, suicide, post-traumatic stress disorder, intimate partner violence, and maternal and infant mortality. | Resources should be directed to the sexual and reproductive health workforce and ensure access to skilled health workers for deliveries and emergency obstetric care. Telemedicine can be used to provide access to medication abortion, contraception and STI prevention. |
| **COVID-19 and reproductive justice in Great Britain and the United States: ensuring access to abortion care during a global pandemic /** Romanis, E. C., Parsons, J. A., Hodson, N. / 2020[49] | UK, USA / Journal Article | Clinic closures, changes in legislation, have caused women seeking an abortion to travel further than before. Increased access via telemedicine has helped somewhat to plug this gap. | Increased inequities in access to abortion, and increased risk of contracting COVID-19 while seeking such services | Protect access to abortion and contraception even during a pandemic to reduce risks posed to women seeking such services |

**Table S2: Abortion Legislation in Africa[50]**

| **Country/Countries** | **Change in Abortion Legislation (if none, what is current status of abortion)** |
| --- | --- |
| Algeria, Gambia, Sierra Leona | No change, only to save life of woman or preserve her physical/mental health. |
| Angola, Congo-Brazzaville, Congo-Kinshasa, Egypt, Gabon, Guinea-Bissau, Madagascar, Mauritania, São Tomé and Príncipe, Senegal | No change, completely prohibited. |
| Botswana, Eritrea, Ghana, Liberia, Mauritius, Mozambique, Namibia, Seychelles, Swaziland | No change, only to save life of woman or preserve her physical/mental health, with exceptions for rape, incest or foetal anomaly. |
| Benin, Burkina Faso, Central African Republic, Ethiopia, Guinea, Lesotho, Rwanda, Togo, Zimbabwe | No change, only to save life of woman or preserve her physical health with exceptions for rape, incest or foetal anomaly. |
| Burundi, Comoros, Djibouti, Equatorial Guinea, Kenya, Morocco | No change, only to save life of woman or preserve her physical health. |
| Cabo Verde | No change, legal on request up to 12 weeks, after 12 weeks of gestation, only for risk to physical or mental health, or foetal anomaly, rape, incest.[51] |
| Cameroon | No change, only to save life of woman or preserve her physical health with exceptions for rape. |
| Chad, Niger | No change, only to save life of woman or preserve her physical health with exceptions for foetal anomaly. |
| Côte d’Ivoire, Libya, Malawi, Nigeria, Somalia, South Sudan, Tanzania, Uganda | No change, only to save life of woman. |
| Eritrea | No change, only to save life of woman or preserve her physical/mental health, with exceptions for rape or incest. |
| Mali | No change, only to save life of woman, with exceptions for rape and incest. |
| South Africa | TEMA was previously prohibited unless the provider and patient had a prior relationship, but this regulation has been relaxed due to COVID-19.[52]  Legal on request up to 13 weeks. From 13 to 20 weeks, only for woman’s physical/mental health, in cases of foetal anomaly, rape, incest or socioeconomic reasons. After 20 weeks, only if maternal or foetal life in danger, or there are serious birth defects.[53] |
| Sudan | No change, only to save life of woman, with exceptions for incest. |
| Tunisia | No change, abortion on request up to 3 months of gestation with exceptions for foetal anomalies and threats to maternal life.[54] |
| Zambia | No change, only permitted to save life of woman, preserve physical or mental health, socio- economic reasons, or for cases of foetal anomaly. |

**Table S3: Abortion Legislation in Asia[55]**

| **Country/Countries** | **Change in Abortion Legislation (if none, what is current status of abortion)** |
| --- | --- |
| Afghanistan, Bangladesh, Brunei Darussalam, Lebanon, Myanmar, Oman, Sri Lanka, Syria, Timor Leste, United Arab Emirates, West Bank and Gaza, Yemen | No change, only to save the mother’s life. |
| Armenia | No change, legal on request up to 12 weeks.[56] |
| Azerbaijan | No change, legal on request up to 12 weeks, and in special circumstances from 13-28 weeks.[57] |
| Bahrain | No change, legal on request, upon authorisation from a panel of three doctors.[58] Only illegal if self-induced. |
| Bhutan | No change, only to save the mother’s life.  Exceptions for rape and incest. |
| China | No change.  **Hong Kong**: Only when performed by registered doctors at gazetted hospitals or Family Planning Association Hong Kong (FPAHK), and the pregnancy is less than 10 weeks of gestation.[59]  **Mainland China**: Available on request if the mother has a pre-existing medical condition causing threat to maternal life, when the mother has already undergone two or more Caesarean sections, expected spontaneous abortion, women working in certain occupations, women with more than 4 children, or who had given birth four months prior.[60] |
| Cambodia | No change, require supervision by a medical professional in a hospital, health centre, or clinic which is authorised by the Ministry for Public Health, which must have the capacity to provide emergency medical treatment and means of transport to hospital.[61]  Allowed up to 10 weeks for medical and 12 weeks for surgical abortions. |
| Cyprus, Japan | No change, only to save life of woman, preserve physical or mental health or for socioeconomic reasons with exception for rape in both countries and foetal anomaly in Cyprus. |
| India | Changed from 20 to 24 weeks except in cases of foetal anomaly, rape, incest or statutory rape.[62] |
| Indonesia | No change, only to save the mother’s life.  Exceptions for rape and foetal anomaly. |
| Iran | No change, only to save the mother’s life.  Exceptions for foetal anomaly. |
| Iraq, Laos, The Philippines | No change, not permitted for any reason. |
| Israel, Malaysia | No change, only for mother’s life, or physical or mental health.  Exceptions for rape, incest and foetal anomaly in Israel. |
| Jordan, Kuwait, Maldives, Pakistan, Qatar, Saudi Arabia | No change, only for mother’s life or physical health, with exceptions for foetal anomaly in Kuwait and Qatar. |
| Kazakhstan | No change, up to 12 weeks on request.  From 12-22 weeks only in certain cases, and no time limit if there are medical indications threatening the life of the woman, but the woman must consent.[57] |
| Kyrgyzstan | No change, allowed up to 12 weeks on request, 22 weeks for “social reasons”.[63] |
| Mongolia | No change, available on request up to the first trimester. Second trimester abortion is subject to approval of the Medical Committee. Late-term abortions are permitted only in certain scenarios such as rape, incest, maternal mental disorder, to save the life/health of the mother/foetus, at request if mother above 45 or below 16.[64] |
| Nepal | No change, legal up to 12 weeks of gestation, and 18 weeks for cases of rape or incest, and at any stage if physical/mental health of the woman is threatened, or if there are severe foetal anomalies.[65] |
| North Korea | No change, permitted for “important reasons”, which have not been specified.[66] |
| South Korea | No change due to COVID-19.  Proposed changes to current laws to allow abortions up to a maximum of 14 weeks, with extension up to 24 weeks for the mother’s health, severe birth defects or if they have been the victim of a rape (as of Nov 2020).[67] |
| Singapore | No change, voluntary abortion allowed up to 24 weeks of gestation, only surgical abortions performed by an authorised medical practitioner permitted from 16-24 weeks.[68] |
| Taiwan | No change, allowed up to 24 weeks for specific reasons (medical reasons, rape, incest, psychiatric illness), spousal or parental consent required.[69] |
| Thailand | Previously only for mother’s life, or physical or mental health.  Will be permitted up to 12 weeks of pregnancy for any reason, with exceptions including severe foetal impairment, medical conditions, risk to mother’s life, cases of rape, deception or coercion as of 7 February 2021.[70] |
| Tajikistan | No change, allowed on request up to 12 weeks, and on certain grounds after 12 weeks, with no time limit if the life of the woman is endangered.[57] |
| Turkmenistan | No change, legal on request up to 12 weeks, from 12-28 weeks allowed for a variety of reasons with approval from a commission of local doctors.[71] |
| Uzbekistan | No change, allowed on request up to 12 weeks, and on medical or social grounds during the second trimester.[72] |
| Vietnam | No change, allowed from 6 to 22 weeks dependent on setting.[73] |

**Table S4: Abortion Legislation in Europe[47]**

| **Country/Countries** | **Change in Abortion Legislation (if none, what is current status of abortion)** |
| --- | --- |
| Albania | No change, gestational limit of 12 weeks for non-medical indications. |
| Andorra | No change, ban on elective abortions. |
| Austria | No change, gestational limit of 16 weeks for non-medical indications. |
| Belarus | No change, gestational limit of 12 weeks for non-medical indications. |
| Belgium | Changes in abortion care depended on facility type and region. While outpatient clinics continued to provide abortion care, some hospitals stopped offering abortion care or reduced facility-based abortion by promotion medical abortions up to 9 weeks + 6 days with home use of misoprostol. |
| Bosnia and Herzegovina | No change, allowed up to 10 weeks on request. Must be approved by a committee and will be permitted for cases of foetal impairment, rape, incest, and for the woman’s mental or physical health up to 20 weeks. After 20 weeks, abortion may be permitted to save the life or health of the woman. All women must first undergo counselling.[57] |
| Bulgaria | No change, gestational limit of 12 weeks for non-medical indications. |
| Croatia | No change, gestational limit of 10 weeks for non-medical indications. |
| Cyprus | No change, gestational limit of 12 weeks for non-medical indications. |
| Czech Republic | No change, gestational limit of 7 weeks for non-medical indications. |
| Denmark (including Greenland) | No change, gestational limit of 12 weeks for non-medical indications. |
| Estonia | Remote consultation by phone is now allowed and use of misoprostol at home is encouraged (this was also available before COVID-19). |
| Finland | Home use of misoprostol extended up to 10 weeks + 0 days (previously 9 weeks + 0 days in Helsinki. |
| France | Gestational limit of medical abortion at home raised from 7 weeks to 9 weeks + 0 days. Medical abortions all performed by telemedicine without mandatory in person consultation. |
| Georgia | No change, gestational limit of 12 weeks for non-medical indications. |
| Germany | Mandatory counselling can be performed via telemedicine, but most abortions are performed surgically. |
| Greece | No change, available on request up to 12 weeks. Permitted up to 19 weeks in the case of rape or incest, and 24 weeks in the case of severe foetal anomalies.[74] |
| Hungary | In the public sector, surgical methods of abortion, which are the only method available in Hungary, were seized due to government ban on non-life saving procedures.  In private sector, abortion was unavailable due to the closure of most private clinics. |
| Iceland | No change, gestational limit of 22 weeks for non-medical indications. |
| Italy | Postponement of non-urgent procedures mandated.  Medical: up to 7 weeks  Surgical: up to 90 days |
| Republic of Ireland | No change, available on request up to 12 weeks, and later in cases of foetal anomalies, or threats to the woman’s life or health. |
| Latvia | No change, gestational limit of 12 weeks for non-medical indications. |
| Liechtenstein | No change, ban on elective abortions. |
| Lithuania | No change, gestational limit of 12 weeks for non-medical indications. |
| Luxembourg | No change, gestational limit of 14 weeks for non-medical indications. |
| Malta | No change, total ban on abortion. |
| Moldova | No change, allowed up to 12 weeks on request. Permitted up to 22 weeks in cases of rape, incest, foetal anomalies, socioeconomic reasons, or for the woman’s health and up to 28 weeks in cases of severe foetal malformation or congenital syphilis.[57] |
| Montenegro | No change, gestational limit of 10 weeks for non-medical indications. |
| Monaco | No change, ban on elective abortions. |
| Netherlands | No change, gestational limit of 22 weeks for non-medical indications. |
| North Macedonia | No change, legal on request up to 12 weeks. Permitted up to 22 weeks in cases of rape, incest, foetal anomalies, socioeconomic reasons or for the mental or physical health of the mother.[75] |
| Norway | There is an introduction of non-facility-based abortion by allowing gynaecologists outside of hospitals to conduct abortion up to 12 weeks’ gestation. (pre-COVID, abortions were only facility-based). |
| Poland | Abortion is now only permitted in cases of rape, incest, or to save the life of the woman under a new ruling by the Constitutional Court in October 2020, which took effect in January 2021. No longer permitted in cases of foetal anomalies.[76] |
| Portugal | Waiting period is omitted, only one visit with a doctor for ultrasound and abortion needed. Follow-up visit can be done by telemedicine or postponed. |
| Romania | No change, gestational limit of 14 weeks for non-medical indications. |
| Russian Federation | No change, gestational limit of 12 weeks for non-medical indications. |
| San Marino | No change, ban on elective abortions. |
| Serbia | No change, gestational limit of 10 weeks for non-medical indications. |
| Slovakia | No change, gestational limit of 12 weeks for non-medical indications. |
| Slovenia | No change, gestational limit of 10 weeks for non-medical indications. |
| Spain | No change, gestational limit of 14 weeks for non-medical indications. |
| Sweden | No change, gestational limit of 18 weeks for non-medical indications. |
| Switzerland | Some clinics have extended the gestational age limit for medical abortion from 7 to 9 weeks due to difficulties accessing surgical abortion. |
| Ukraine | No change, available on request up to 12 weeks’ gestation. Permitted up to 22 weeks in cases of rape, incest, foetal anomalies, for the woman’s physical or mental health, socioeconomic reasons, and if the woman is intellectually or cognitively disabled^92^. |
| United Kingdom (Northern Ireland) | Revised model of care during the COVID-19 pandemic which provides for remote consultation with a doctor for the purpose of accessing termination in the early pregnancy. Face-to-face consultation was previously needed to physically examine the pregnant woman before a decision of abortion can be made.[77] Abortion services started to operate in April 2020 for first trimester abortions. |
| United Kingdom (Scotland) | In March 2020, due to the COVID-19 outbreak in UK, medical abortion (mifepristone) at home by telemedicine for pregnancies at less than 12 weeks’ gestation is now permitted.[26] |
| United Kingdom (Wales and England) | Women are permitted to have medical abortions prescribed via telemedicine provided that the pregnancy is under 9 weeks and 6 days, valid until March 21, 2022. |

**Table S5: Abortion Legislation in Oceania**

| **Country/Countries** | **Change in Abortion Legislation (if none, what is current status of abortion)** |
| --- | --- |
| Australia | No change.  **Australian Capital Territory**: Abolition of Offence of Abortion Act 2002  **NSW**: Abortion Law Reform Act 2019 (Up till 22 weeks of gestation or later if 2 doctors agree).  **Queensland**: Termination of Pregnancy Bill 2018 (Up till 22 weeks of gestation or later if 2 doctors agree).  **South Australia:** The Termination of Pregnancy Bill passed the state’s upper house in March 202, thus abortion will be moved out of the criminal code and regulated under health law. Abortions can now be provided beyond certain hospitals and women no longer need to have been South Australia residents for 2 months or more to access abortion.  The gestational limit for an “on request” abortion in South Australia is now 22 weeks and six days.[78]  **Tasmania**: Reproductive Health Act 2013 (Up till 16 weeks of gestation or later if 2 doctors agree).  **Northern Territory**: Termination of Pregnancy Law Reform Bill 2017 (Up till 14 weeks of gestation if a doctor deems appropriate. Between 14 to 23 weeks of gestation, if 2 doctors deem appropriate).  **Western Australia**: Acts Amendment Abortion Act 1998 (Up till 20 weeks of gestation or later if two doctors agree).  **Victoria**: Abortion Law Reform Act 2008 (Up till 24 weeks of gestation or later if 2 doctors agree). |
| Cook Islands, Vanuatu | No change, only legal to save life of woman, preserve physical or mental health.[79, 80] |
| Fiji | No change, only legal to save life of woman, preserve physical or mental health, and if pregnancy is as a result of rape or incest.[81] |
| Kiribati, Nauru, Niue, Papua New Guinea, Solomon Islands, Tuvalu | No change, only legal to save life of woman. [82-87] |
| Micronesia | No change, not clear if abortion is a crime.[88] |
| New Zealand | Legalised as of March 2020 up till 20 weeks of gestation or later if agreed by a doctor.[89] |
| Samoa | No change, only legal to save life of woman or preserve physical or mental health if less than 20 weeks of gestation.[90] |
| Tonga | No change, legal only to preserve maternal health.[91] |

**Table S6: Abortion Legislation in North America**

| **Country/Countries** | **Change in Abortion Legislation (if none, what is current status of abortion)** |
| --- | --- |
| Canada | No change, legal at any stage of pregnancy from first to third trimester, before live birth, regardless of reason.[92] |
| United States of America, The | **Arizona, Arkansas, Indiana, South Carolina, and West Virginia** have previously banned TEMA.[46]  **Texas, Mississippi and Ohio** have passed bans on TEMA this year as well.  **Alabama, Oklahoma, Louisiana, Mississippi, Missouri, Nebraska, North Carolina, North Dakota, South Dakota, Tennessee, Texas, and Wisconsin** already had laws mandating the presence of a physician during an abortion, which would make TEMA unlawful.[49]  **Ohio, Texas, Louisiana, Mississippi, Maryland, Tennessee, Kentucky, Iowa, Oklahoma, Indiana & West** **Virginia:** Declared all elective abortions as non-essential and to be ceased.[25]  **All other states:** No change, legal. |

**Table S7: Abortion Legislation in Latin America & The Caribbean [93]**

| **Country/Countries** | **Change in Abortion Legislation (if none, what is current status of abortion)** |
| --- | --- |
| Antigua and Barbuda, Dominica | No change, only to save the life of the woman. |
| Argentina | Legalisation of abortion delayed due to COVID-19.[94]  Legal up to 14 weeks as of 27 December 2020.[95] |
| Bahamas, Costa Rica, Ecuador, Grenada, Peru | No change, to save life of woman or preserve physical health. |
| Barbados, St. Vincent and Grenadines | No change, to save life of woman, for physical/mental health, socioeconomic reasons, in cases of foetal anomaly, or rape and incest. |
| Belize | No change, to save life of woman, for physical/mental health, socioeconomic reasons, in cases of foetal anomaly. |
| Bolivia | No change, allowed only to save the mother's life or health, and in cases of rape. |
| Brazil | No change, to save life of woman or in cases of rape. |
| Chile, Mexico, Panama | No change, allowed only to save the mother’s life, in cases of rape, and foetal malformation. |
| Colombia | No change, illegal unless the pregnancy is the result of rape, abusive sexual intercourse without consent, incest and artificial insemination or transfer of a fertilized ovum without consent.[96] |
| Cuba | No change, available on request up to 12 weeks of gestation. From 12-22 weeks, exceptions only for medical reasons or for health. From 22-26 weeks, exceptions for genetic defects. From 26-35 weeks, exceptions for exceptional reasons relating to genetic causes.[97] |
| Dominican Republic, El Salvador, Haiti, Honduras, Nicaragua, Suriname | No change, completely prohibited. |
| Ecuador | No change, allowed only to save the mother's life or health, or in cases of rape of disabled women. |
| French Guiana | As according to the French Penal Code. |
| Guatemala, Paraguay, Venezuela | No change, allowed only to save the mother's life. |
| Guyana | No change. Legal up to 8 weeks of gestation on request, under medical supervision in any setting. From 8-12 weeks, can be performed by a doctor or a doctor’s assistant in an approved institution. From 12-16 weeks, can be performed by a medical practitioner in an approved institution, after approval of two doctors. After 16 weeks, only if three doctors believe that the abortion must be performed to save the mother’s life or health, or foetal health. Requires 48 hours between counselling and abortion.[98] |
| Jamaica, St. Kitts and Nevis, Trinidad and Tobago | No change, to save life of woman, or for physical/mental health. |
| Mexico | No change, abortion is legal only in Mexico City and Oaxaca up to 12 weeks of gestation, with other states having exceptions for rape, incest, socioeconomic reasons, foetal anomalies.[99] |
| Puerto Rico | No change, legal on request at any point in the pregnancy (although most still believe it is illegal).[100] |
| Saint Lucia | No change, to save life of woman, or for physical/mental health, or in cases of rape or incest. |
| Uruguay | No change, allowed on request up to 12 weeks of gestation, 14 weeks in cases of rape, and no time limit if woman’s health is at risk or in cases of foetal anomaly.[101] |

**REFERENCES**

1. Dahlen HG, Kumar-Hazard B, Chiarella M. How COVID-19 Highlights an Ongoing Pandemic of Neglect and Oppression When It Comes to Women's Reproductive Rights. J Law Med. 2020;27(4):812-28.

2. Bateson DJ, Lohr PA, Norman WV, Moreau C, Gemzell-Danielsson K, Blumenthal PD, et al. The impact of COVID-19 on contraception and abortion care policy and practice: experiences from selected countries. BMJ Sex Reprod Health. 2020;46(4):241-3.

3. Trapani Junior A, Vanhoni LR, Silveira SK, Marcolin AC. Childbirth, Puerperium and Abortion Care Protocol during the COVID-19 Pandemic. Rev Bras Ginecol Obstet. 2020;42(6):349-55.

4. Todd-Gher J, Shah PK. Abortion in the context of COVID-19: a human rights imperative. Sex Reprod Health Matters. 2020;28(1):1758394.

5. Assis MP, Larrea S. Why self-managed abortion is so much more than a provisional solution for times of pandemic. Sex Reprod Health Matters. 2020;28(1):1779633.

6. Zhang L, Jiang Y, Wei M, Cheng BH, Zhou XC, Li J, et al. [Analysis of the pregnancy outcomes in pregnant women with COVID-19 in Hubei Province]. Zhonghua Fu Chan Ke Za Zhi. 2020;55(3):166-71.

7. Li G, Tang D, Song B, Wang C, Qunshan S, Xu C, et al. Impact of the COVID-19 Pandemic on Partner Relationships and Sexual and Reproductive Health: Cross-Sectional, Online Survey Study. J Med Internet Res. 2020;22(8):e20961.

8. Wu YT, Li C, Zhang CJ, Huang HF. Is termination of early pregnancy indicated in women with COVID-19? Eur J Obstet Gynecol Reprod Biol. 2020;251:271-2.

9. Wang Y, Yang Q. Post Abortion Care and Management After Induced Abortion During the COVID-19 Pandemic: A Chinese Expert Consensus. Adv Ther. 2021;38(2):1011-23.

10. Liu D, Li L, Wu X, Zheng D, Wang J, Yang L, et al. Pregnancy and Perinatal Outcomes of Women With Coronavirus Disease (COVID-19) Pneumonia: A Preliminary Analysis. AJR Am J Roentgenol. 2020;215(1):127-32.

11. Tang K, Gaoshan J, Ahonsi B, Ali M, Bonet M, Broutet N, et al. Sexual and reproductive health (SRH): a key issue in the emergency response to the coronavirus disease (COVID- 19) outbreak. Reprod Health. 2020;17(1):59.

12. Sahoo S, Pattnaik JI, Mehra A, Nehra R, Padhy SK, Grover S. Beliefs related to sexual intimacy, pregnancy and breastfeeding in the public during COVID-19 era: a web-based survey from India. J Psychosom Obstet Gynaecol. 2020:1-8.

13. Kumar N. COVID 19 era: a beginning of upsurge in unwanted pregnancies, unmet need for contraception and other women related issues. Eur J Contracept Reprod Health Care. 2020;25(4):323-5.

14. Sharma KA, Zangmo R, Kumari A, Roy KK, Bharti J. Family planning and abortion services in COVID 19 pandemic. Taiwan J Obstet Gynecol. 2020;59(6):808-11.

15. Vora KS, Saiyed S, Natesan S. Impact of COVID-19 on family planning services in India. Sex Reprod Health Matters. 2020;28(1):1785378.

16. Chandrasekaran S, Diamond-Smith N, Srinivasan K, Dalvie S. Preparing for an Increased Need for Abortion Access in India during and after COVID-19: Challenges and Strategies. Stud Fam Plann. 2020;51(4):377-83.

17. Gibelin K, Agostini A, Marcot M, Piclet H, Bretelle F, Miquel L. COVID-19 impact in abortions' practice, a regional French evaluation. J Gynecol Obstet Hum Reprod. 2021;50(5):102038.

18. Cioffi A, Cioffi F, Rinaldi R. COVID-19 and abortion: The importance of guaranteeing a fundamental right. Sex Reprod Healthc. 2020;25:100538.

19. Bellizzi S, Ronzoni AR, Pichierri G, Cegolon L, Salaris P, Panu Napodano CM, et al. Safe abortion amid the COVID-19 pandemic: The case of Italy. Int J Gynaecol Obstet. 2020;150(2):254-5.

20. Caruana-Finkel L. Abortion in the time of COVID-19: perspectives from Malta. Sex Reprod Health Matters. 2020;28(1):1780679.

21. Kumar M, Daly M, De Plecker E, Jamet C, McRae M, Markham A, et al. Now is the time: a call for increased access to contraception and safe abortion care during the COVID-19 pandemic. BMJ Glob Health. 2020;5(7).

22. Church K, Gassner J, Elliott M. Reproductive health under COVID-19 - challenges of responding in a global crisis. Sex Reprod Health Matters. 2020;28(1):1-3.

23. Gujski M, Humeniuk E, Bojar I. Current State of Knowledge About SARS-CoV-2 and COVID-19 Disease in Pregnant Women. Med Sci Monit. 2020;26:e924725.

24. Aiken A, Lohr PA, Lord J, Ghosh N, Starling J. Effectiveness, safety and acceptability of no-test medical abortion (termination of pregnancy) provided via telemedicine: a national cohort study. BJOG. 2021.

25. Romanis EC, Parsons JA. Legal and policy responses to the delivery of abortion care during COVID-19. Int J Gynaecol Obstet. 2020;151(3):479-86.

26. Reynolds-Wright JJ, Johnstone A, McCabe K, Evans E, Cameron S. Telemedicine medical abortion at home under 12 weeks' gestation: a prospective observational cohort study during the COVID-19 pandemic. BMJ Sex Reprod Health. 2021.

27. Wenham C, Smith J, Davies SE, Feng H, Grepin KA, Harman S, et al. Women are most affected by pandemics - lessons from past outbreaks. Nature. 2020;583(7815):194-8.

28. Bayefsky MJ, Bartz D, Watson KL. Abortion during the Covid-19 Pandemic - Ensuring Access to an Essential Health Service. N Engl J Med. 2020;382(19):e47.

29. Ruggiero S, Brandi K, Mark A, Paul M, Reeves MF, Schalit O, et al. Access to later abortion in the United States during COVID-19: challenges and recommendations from providers, advocates, and researchers. Sex Reprod Health Matters. 2020;28(1):1774185.

30. Fang NZ, Castano PM, Davis A. A hospital-based COVID-19 abortion case in the early phase of the pandemic. Contraception. 2020;102(2):137-8.

31. White K, Kumar B, Goyal V, Wallace R, Roberts SCM, Grossman D. Changes in Abortion in Texas Following an Executive Order Ban During the Coronavirus Pandemic. JAMA. 2021;325(7):691-3.

32. Tanne JH. Coronavirus pandemic stirs fight over abortion rights in US. BMJ. 2020;369:m1733.

33. Jones RK, Lindberg L, Witwer E. COVID-19 Abortion Bans and Their Implications for Public Health. Perspect Sex Reprod Health. 2020;52(2):65-8.

34. Roberts SCM, Schroeder R, Joffe C. COVID‐19 and Independent Abortion Providers: Findings from a Rapid‐Response Survey. Perspectives on Sexual and Reproductive Health. 2020;52(4):217-25.

35. Andersen M, Bryan S, Slusky D. COVID-19 Surgical Abortion Restriction Did Not Reduce Visits to Abortion Clinics2020.

36. Tanne JH. Covid-19: Women's health campaigners sue FDA over access to medical abortion pills. BMJ. 2020;369:m2187.

37. Aiken ARA, Starling JE, Gomperts R, Tec M, Scott JG, Aiken CE. Demand for Self-Managed Online Telemedicine Abortion in the United States During the Coronavirus Disease 2019 (COVID-19) Pandemic. Obstet Gynecol. 2020;136(4):835-7.

38. Gambir K, Garnsey C, Necastro KA, Ngo TD. Effectiveness, safety and acceptability of medical abortion at home versus in the clinic: a systematic review and meta-analysis in response to COVID-19. BMJ Glob Health. 2020;5(12).

39. Ahmed A. How the COVID-19 response is altering the legal and regulatory landscape on abortion. J Law Biosci. 2020;7(1):lsaa012.

40. Baill IC. Medical Abortion Care During a Pandemic. J Patient Exp. 2020;7(3):291-2.

41. Robinson EF, Moulder JK, Zerden ML, Miller AM, Zite NB. Preserving and advocating for essential care for women during the coronavirus disease 2019 pandemic. Am J Obstet Gynecol. 2020;223(2):219-20 e1.

42. Sackeim MG. Protecting Access To Abortion During The COVID-19 Pandemic. Health Aff (Millwood). 2020;39(8):1456-8.

43. McSpedon C. Reproductive Care During COVID-19. Am J Nurs. 2020;120(9):19-20.

44. Gross MS, Harrington BJ, Sufrin CB, Faden RR. Rethinking "Elective" Procedures for Women's Reproduction during Covid-19. Hastings Cent Rep. 2020;50(3):40-3.

45. Cohen MA, Powell AM, Coleman JS, Keller JM, Livingston A, Anderson JR. Special ambulatory gynecologic considerations in the era of coronavirus disease 2019 (COVID-19) and implications for future practice. Am J Obstet Gynecol. 2020;223(3):372-8.

46. Donley G, Chen BA, Borrero S. The legal and medical necessity of abortion care amid the COVID-19 pandemic. J Law Biosci. 2020;7(1):lsaa013.

47. Moreau C, Shankar M, Glasier A, Cameron S, Gemzell-Danielsson K. Abortion regulation in Europe in the era of COVID-19: a spectrum of policy responses. BMJ Sex Reprod Health. 2020.

48. Hall KS, Samari G, Garbers S, Casey SE, Diallo DD, Orcutt M, et al. Centring sexual and reproductive health and justice in the global COVID-19 response. The Lancet. 2020;395(10231):1175-7.

49. Romanis EC, Parsons JA, Hodson N. COVID-19 and reproductive justice in Great Britain and the United States: ensuring access to abortion care during a global pandemic. J Law Biosci. 2020;7(1):lsaa027.

50. The Guttmacher Institute. FACT SHEET: ABORTION IN AFRICA: The Guttmacher Institute; 2018 [updated March 2018. Available from: https://www.guttmacher.org/sites/default/files/factsheet/ib_aww-africa.pdf.

51. WHO. Country Profile: Cabo Verde: World Health Organisation, Human Reproduction Programme; 2020 [Available from: https://abortion-policies.srhr.org/country/cabo-verde/.

52. Kwinda M. Notice to amend Telemedicine Guidelines during COVID-19 – dated 3 April 2020: Health Professions Council of South Africa; 2020 [cited 2021 January 2]. Available from: https://www.hpcsa-blogs.co.za/notice-to-amend-telemedicine-guidelines-during-covid-19/.

53. CHOICE ON TERMINATION OF PREGNANCY ACT, (18 February 2008, 2008).

54. WHO. Country Profile: Tunisia: World Health Organisation, Human Reproduction Programme; 2017 [7 May 2017:[Available from: https://abortion-policies.srhr.org/country/tunisia/.

55. The Guttmacher Institute. FACT SHEET: ABORTION IN ASIA: The Guttmacher Institute; 2018 [cited 2020 December 29]. Available from: https://www.guttmacher.org/sites/default/files/factsheet/ib_aww-asia.pdf.

56. WHO. Country Profile: Armenia: World Health Organisation, Human Reproduction Programme; 2017 [May 7, 2017:[Available from: https://abortion-policies.srhr.org/country/armenia/.

57. United Nations Department of Economic and Social Affairs Population Division. Abortion Policies and Reproductive Health around the World. United Nations Publications [Internet]. 2014 January 2, 2021. Available from: https://www.un.org/en/development/desa/population/publications/pdf/policy/AbortionPoliciesReproductiveHealth.pdf.

58. Legislative Decree No (7) for 1989 On the Practice of Human Medicine and Dentistry, (1989).

59. Family Health Service. Termination of pregnancy. In: Department of Health, editor. Hong Kong Special Administrative Region2016.

60. Luk BH-k. Abortion in Chinese Law. The American Journal of Comparative Law. 1977;25(2):372-92.

61. Law on Abortion, Article 6 (1997).

62. The Medical Termination of Pregnancy (Amendment) Bill, 2020, (2020).

63. Law No.148 of 4 July 2015 "On Reproductive Rights of Citizens and Guarantees of their Realization". Article 16 (2015).

64. Health Law of Mongolia, Article 36 (1998).

65. 11th Amendment to the Civil Code, (2002).

66. Criminal Code of March 1950, (1950).

67. Cha S. South Korea proposes compromise abortion law after landmark court ruling. Reuters. 2020 October 7, 2020.

68. TERMINATION OF PREGNANCY ACT (Chapter 324), (1987).

69. Genetic Health Act, (2009).

70. Abortion laws to be amended by court ruling. The Bangkok Post [Internet]. 2021 30 April 2021 [cited 2021 April 30]. Available from: https://www.bangkokpost.com/thailand/general/1861914/abortion-laws-to-be-amended-by-court-ruling.

71. WHO. Country Profile: Turkmenistan: World Health Organisation, Human Reproduction Programme; 2017 [Available from: https://abortion-policies.srhr.org/country/turkmenistan/.

72. WHO. Country Profile: Uzbekistan: World Health Organisation, Human Reproduction Programme; 2017 [Available from: https://abortion-policies.srhr.org/country/uzbekistan/.

73. WHO. Country Profile: Vietnam: World Health Organisation, Human Reproduction Programme; 2017 [Available from: https://abortion-policies.srhr.org/country/vietnam/.

74. Europe's abortion rules. BBC News. 2007 12 February 2007.

75. IPPF EN. North Macedonia’s abortion care law signals new dawn for reproductive freedom: International Planned Parenthood Federation European Network; 2019 [updated 4 April; cited 2021 18 May 2021]. Available from: https://www.ippfen.org/blogs/north-macedonias-abortion-care-law-signals-new-dawn-reproductive-freedom-0.

76. Poland enforces controversial near-total abortion ban. BBC News. 2021 28 January 2021.

77. Conneely A. Changes in abortion service due to Covid-19. RTÉ [Internet]. 2020 December 29, 2020. Available from: https://www.rte.ie/news/2020/0408/1129326-interim-model-sees-abortion-care-take-place-remotely/.

78. Millar E, Baird B. Abortion is no longer a crime in Australia. But legal hurdles to access remain. The Conversation. 2021 4 March 2021.

79. Crimes Act 1969. Sect. 63, 202-207 (1969).

80. Penal Code [Cap 135] LAWS OF THE REPUBLIC OF VANUATU CONSOLIDATED EDITION 2006, Act No. 17 of 7 August 1981. Sect. 117 (2006).

81. Penal Code.

82. Penal Code, LAWS OF THE GILBERT ISLANDS, (1977).

83. Crimes Act 2016. Sect. 224-226 (2016).

84. Part V of the Niue Act 1966, (1966).

85. McGoldrick IA. Termination of pregnancy in Papua New Guinea: the traditional and contemporary position. Papua and New Guinea medical journal. 1981;24(2):113-20.

86. Revised Laws of the Solomon Islands 1996, (1966).

87. Penal Code. Sect. 152 (2008).

88. Murgatroyd P. Abortion Policies: a review of policies and legislation for the South Pacific: University of the South Pacific; 2000 [cited 2021 January 2]. Available from: https://www.usp.ac.fj/index.php?id=15964.

89. New Zealand passes law decriminalising abortion. BBC News. 2020 18 March 2020.

90. Crimes Amendment Act, Article 73 (1969).

91. Criminal Offences Act, Sections 103-105 (2016).

92. Long L, Foot R, McIntosh A. Abortion in Canada2020 January 2, 2021 [cited 2021 January 2]. Available from: https://www.thecanadianencyclopedia.ca/en/article/abortion.

93. The Guttmacher Institute. FACT SHEET: ABORTION IN LATIN AMERICA AND THE CARIBBEAN: The Guttmacher Institute; 2018 [updated March 2018; cited 2020 December 29, 2020]. Available from: https://www.guttmacher.org/sites/default/files/factsheet/ib_aww-latin-america.pdf.

94. Herrero AH, Mellen R. Argentina just got closer to becoming the largest country in Latin America to legalize abortion. The Washington Post. 2020 December 10, 2020.

95. Watson K. Argentina abortion: Senate approves legalisation in historic decision. BBC News. 2020 30 December 2020.

96. Reuters Staff. Colombia's Constitutional Court rules against legalizing abortion in first 16 weeks of pregnancy. Reuters. 2020 March 3, 2020.

97. RESOLUCION MINISTERIAL No. 24, (2011).

98. MEDICAL TERMINATION OF PREGNANCY ACT 1995, (1995).

99. Mexico Supreme Court rejects state's bid to decriminalise abortion. BBC News. 2020 29 July 2020.

100. Azize-Vargas Y, Avilés LA. Abortion in Puerto Rico: The Limits of Colonial Legality. Reproductive Health Matters. 1997;5(9):56-65.

101. Wood S, Abracinskas L, Correa S, Pecheny M. Reform of abortion law in Uruguay: context, process and lessons learned. Reprod Health Matters. 2016;24(48):102-10.
